# The mechanism of oxythiamine-induced collagen biosynthesis in cultured fibroblasts

**DOI:** 10.1007/s11010-015-2336-z

**Published:** 2015-01-28

**Authors:** Lukasz Szoka, Ewa Karna, Jerzy Palka

**Affiliations:** Department of Medicinal Chemistry, Medical University of Bialystok, Mickiewicza 2 D, 15-222 Bialystok, Poland

**Keywords:** Oxythiamine, Collagen biosynthesis, Prolidase, ERK1/2, Akt, NF-*κ*B p65

## Abstract

The oxythiamine (OXY) is antivitamin of thiamine. The finding that OXY increases the cytoplasmic concentration of pyruvate, known to enhance collagen biosynthesis, led us to investigate the mechanism of this antivitamin action on collagen biosynthesis in cultured human skin fibroblasts. Confluent fibroblasts were treated with micromolar concentrations (30–1,000 µM) of OXY for 24 and 48 h. It was found that OXY-dependent increase in collagen biosynthesis was accompanied by parallel increase in prolidase activity and level, compared to untreated cells. Since phosphoenolpyruvate (PEP) is known as an inhibitor of prolidase—the enzyme that plays important role in collagen biosynthesis, the mechanism of pyruvate interconversion was considered as a regulatory switch in collagen biosynthesis. In fact, 3-MPA, specific inhibitor of phosphoenolpyruvate carboxykinase (PEPCK), contributed to up-regulation of prolidase activity, suggesting that down-regulation of PEP formation is an underlying mechanism. Since collagen biosynthesis and prolidase activity are regulated by signal induced by activated α_2_β_1_ integrin receptor as well as insulin-like growth factor-I receptor (IGF-IR), the expression of these receptors was measured by Western immunoblot analysis. The exposure of the cells to OXY contributed to decrease in IGF-IR, α_2_β_1_ integrin receptor, pERK1/2, and NF-*κ*B p65 expressions. It was accompanied by increase in total ERK1/2 expression and induction of phosphorylation of Akt protein. The data suggest that OXY-dependent increase of collagen biosynthesis in cultured human skin fibroblasts results from activation of prolidase activity and level, induction in pAkt expression and down-regulation of pERK1/2 and NF-*κ*B p65, the known inhibitor of collagen gene expression.

## Introduction

Oxythiamine (OXY) is antivitamin of thiamine. It interferes with the enzymes of thiamine pyrophosphate-dependent pathways and can inhibit conversion of glucose into pentose phosphate that is necessary for the synthesis of nucleotides and metabolism of amino acids [1]. OXY increases the amount of pyruvate by inhibition of pyruvate decarboxylase [2]. Pyruvate has a key position in several metabolic pathways such as glycolysis and gluconeogenesis. Inhibition of pyruvate decarboxylase, which is part of the pyruvate dehydrogenase complex may interfere with the course of the oxidative decarboxylation of pyruvate. The result of this phenomenon is accumulation of pyruvate in the cell.

It was documented that pyruvate enhances collagen biosynthesis in slices of liver of cirrhotic rats [3]. The role of OXY in collagen biosynthesis, however, is not known.

It was also found that in the presence of oxythiamine the activity of cytosolic enzymes, transketolase, and pyruvate decarboxylase was down and up-regulated, dependently on time of incubation. At least in case of pyruvate decarboxylase, increase in the enzyme activity was followed by an increase in the amount of the enzyme protein [4], suggesting transcriptional regulation.

Since PEP is known as an inhibitor of prolidase—the enzyme that play important role in collagen biosynthesis, the mechanism of pyruvate interconversion may represent regulatory switch in collagen biosynthesis.

Prolidase [EC 3.4.13.9] is a cytosolic enzyme that catalyzes the hydrolysis of imidodipeptides with C-terminal proline or hydroxyproline [5–7]. The enzyme plays an important role in the recycling of proline from imidodipeptides (derived from degradation products of collagen) for collagen re-synthesis [8] and cell growth [9]. The efficiency of recycling of proline was found to be about 90 % [10]. It is evident that an absence of prolidase severely impedes the recycling of collagen proline. Some clinical symptoms related to collagen deficit can be attributed to prolidase deficiency [11]. On the other hand, increased activity of liver prolidase was found during fibrotic process [12]. It suggests that the enzyme activity (despite the collagen gene expression) may be a step-limiting factor in regulation of collagen biosynthesis. It has been supported by several studies [13–16].

Prolidase activity is stimulated through a signal mediated by collagen-β_1_ integrin receptor interaction [17, 18]. On the other hand, prolidase activity “in vitro” is inhibited by a strong, competitive inhibitor, PEP, that arises from pyruvate [19, 20].

Another factor that strongly stimulates collagen biosynthesis is insulin-like growth factor-I (IGF-I), acting predominantly through the IGF-I receptor [21, 22]. The effects of IGF-I include induction of collagen gene expression [23], up-regulation of prolidase activity [24], stimulation of mitotic division and prevention of apoptosis [21]. Some of these activities are regulated through NF-*κ*B, the known inhibitor of collagen gene expression [25].

In this study, we examined the effect of OXY on cell viability, collagen biosynthesis, prolidase activity and level, expression of α_2_β_1_ integrin, IGF-I receptor, MAP kinases (ERK_1_, ERK_2_), Akt protein, and the transcription factor—NF-*κ*B p65 in human dermal fibroblasts.

## Materials and methods

Alkaline phosphatase-labeled anti-mouse IgG and anti-rabbit IgG antibodies, bacterial collagenase, 3-(4,5-di-methylthiazole-2-yl)-2,5-diphenyltetrazolium bromide (MTT), Fast BCIP/NBT reagent, l-glycyl-proline, l-proline, monoclonal (mouse) anti-IGF-IR, anti-pERK1/2 and polyclonal (rabbit) anti-β-actin antibodies, sodium pyruvate, and oxythiamine, were provided by Sigma Corp., USA., as were most other chemicals and buffers used. Dulbecco’s minimal essential medium (DMEM) and fetal bovine serum (FBS) used in cell culture were products of Gibco, USA. Glutamine, penicillin, and streptomycin were obtained from Quality Biological Inc., USA. Nitrocellulose membrane (0.2 μm), sodium dodecyl sulphate (SDS), polyacrylamide, molecular weight standards, and Coomassie Brilliant Blue R-250 were received from Bio-Rad Laboratories, USA. L-5[^3^H] proline (28 Ci/mmol) was purchased from Amersham, UK. Monoclonal (mouse) anti-β_1_ and polyclonal (rabbit) anti-α_2_-integrin and anti-NFκB p65 antibodies were the products of Santa Cruz Biotechnology Inc., USA. Polyclonal (rabbit) anti-ERK1/2 and monoclonal (rabbit) anti-pAkt antibodies were the products of Cell Signaling Inc., USA. Polyclonal anti-human prolidase antibody was donated by Dr. James Phang (NCI-Frederick Cancer Research and Development Center, Frederick, MD, USA). 3-mercaptopicolinic acid (3-MPA) was purchased from Applichem GmbH, Germany.

### Tissue culture

All studies were performed on normal human skin fibroblasts (CRL-1474), that were purchased from American Type Culture Collection, Manassas, VA, USA. The cells were maintained in DMEM supplemented with 10 % fetal bovine serum (FBS), 2 mmol/l glutamine, 50 U/ml penicillin, and 50 μg/ml streptomycin at 37 °C in a 5 % CO_2_ incubator. Cells were counted in hemocytometer and cultured at 1 × 10^5^ cells per well in 2 ml of growth medium in 6-well plates (Costar). Cells reached confluence at day 6 and in most cases such cells were used for assays. Cells were used in the 8th to 14th passages.

### Cell viability assay

The assay was performed according to the method of Carmichael [26] using 3-(4,5-di-methylthiazole-2-yl)-2,5-diphenyltetrazolium bromide (MTT). The cells were cultured for 24 and 48 h with various concentrations of OXY in six-well plates, washed three times with PBS, and then incubated for 4 h in 1 ml of MTT solution (0.5 mg/ml of PBS) at 37 °C. The medium was removed, and 1 ml of 0.1 mol/l HCl in absolute isopropanol was added to attached cells. Absorbance of converted dye in living cells was measured at a wavelength of 570 nm. OXY-treated cells viability was calculated as a percent of control cells.

### Determination of prolidase activity

The activity of prolidase was determined according to the method of Myara [27]. Protein concentration was measured by the method of Lowry [28]. Enzyme activity was reported as nanomoles of proline released from synthetic substrate, during 1 min per milligram of supernatant protein of cell homogenate.

### Collagen production

Incorporation of radioactive precursor into proteins was measured after labeling of confluent cells (cultured in growth medium with OXY) for the last 24 h with 5[^3^H] proline (5 μCi/ml, 28 Ci/mM) as described previously [29]. Incorporation of tracer into collagen was determined by digesting proteins with purified *Clostridium histolyticum* collagenase, according to the method of Peterkofsky [30]. Results are shown as combined values for cell plus medium fractions.

### SDS-PAGE

Slab SDS/PAGE was used according to the method of Laemmli [31], using 10 % SDS-polyacrylamide gel.

### Western Immunoblot Analysis

After SDS-PAGE, the gels were allowed to equilibrate for 5 min in 25 mmol/l Tris, 0.2 mol/l glycine in 20 % (v/v) methanol. The protein was transferred to 0.2 μm pore-sized nitrocellulose at 100 mA for 1 h using a LKB 2117 Multiphor II electrophoresis unit. The nitrocellulose was incubated with monoclonal anti-β_1_ and polyclonal anti-α_2_-integrin antibodies at concentration 1:1,000; polyclonal antibodies against NFκB p65, ERK1/2, and β-actin at concentration 1:3,000; polyclonal antibody against prolidase at concentration 1:5,000; monoclonal anti-IGF-IR, pERK1/2 and pAkt antibodies at concentration 1:1,000 in 5 % dried milk in TBS-T (20 mmol/l Tris–HCl buffer, pH 7.4, containing 150 mmol/l NaCl and 0.05 % Tween 20) for 1 h. In order to analyze β_1_ integrin subunit, IGF-IR, and pERK1/2 second antibody, alkaline phosphatase-conjugated anti-mouse IgG (whole molecule) was added at concentration 1:7,500 in TBS-T; in order to analyze prolidase, α_2_-integrin subunit, ERK1/2, pAkt, NFκB p65, and β-actin second antibody, alkaline phosphatase-conjugated anti-rabbit IgG (whole molecule) was added at concentration 1:5,000 in TBS-T and incubated for 30 min slowly shaking. Then nitrocellulose was washed with TBS-T (5 × 5 min) and submitted to Sigma-Fast BCIP/NBT reagent. The intensity of the bands was quantified by densitometric analysis.

### Statistical Analysis

In all experiments, the mean values for three independent experiments done in duplicates ± standard deviation (SD) were calculated. The results were submitted to statistical analysis using the Student’s *t* test, accepting *P* < 0.01 as significant.

## Results

Studies were performed on confluent fibroblasts, since collagen synthesis, prolidase activity as well as IGF-IR expression depend on cell density [32, 33].

**Fig. 1:**
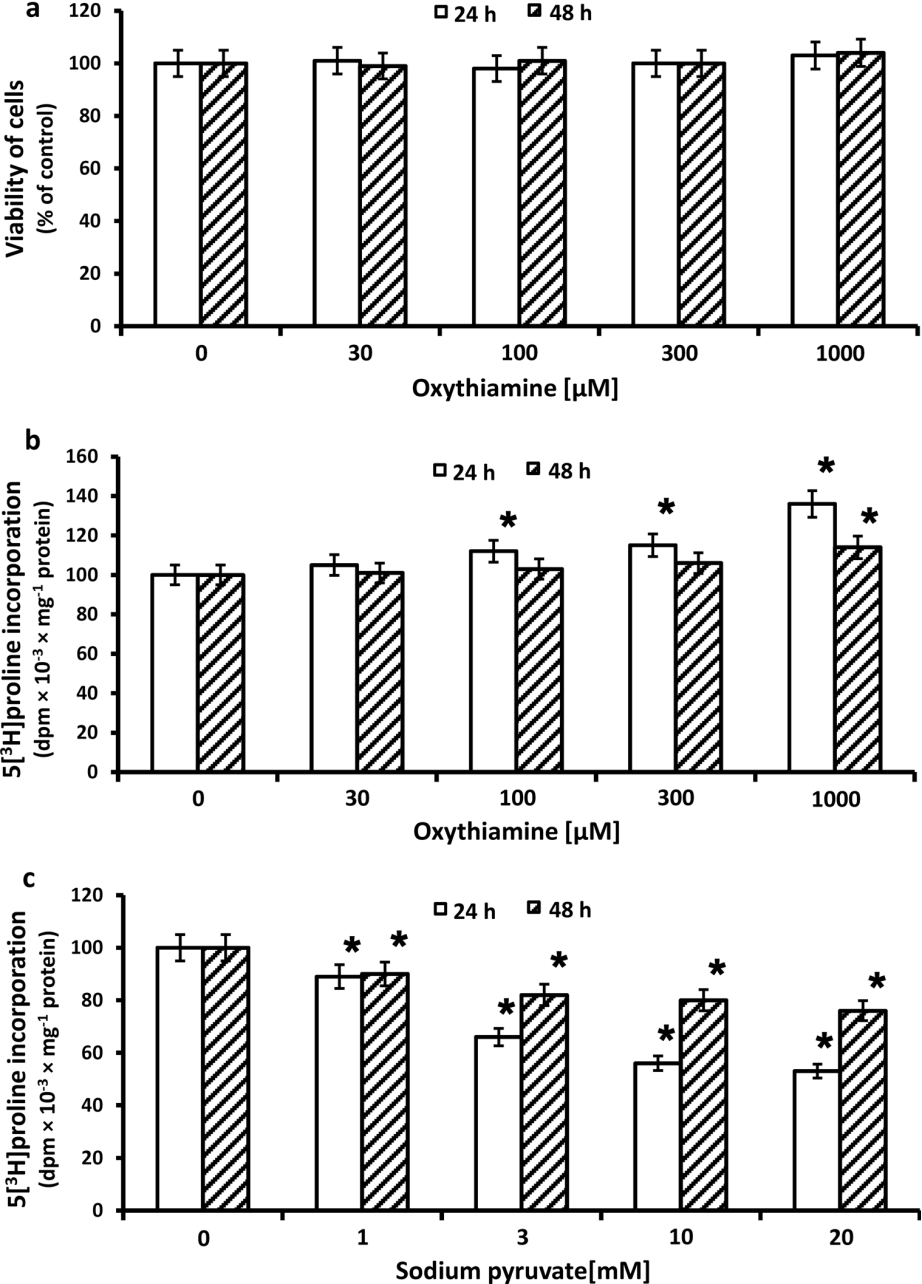
Viability **a** of confluent human skin fibroblasts incubated for 24 and 48 h with different concentrations of oxythiamine (OXY). The mean values ± SD from three independent experiments done in duplicates are presented. *P ≤ 0.01. Collagen biosynthesis measured as 5[^3^H] proline incorporation into proteins susceptible to the action of bacterial collagenase in fibroblasts treated for 24 and 48 h with different concentrations of (OXY) (**b)** and sodium pyruvate (**c**). The results present the mean values from 6 assays ± SD *P ≤ 0.01

Since oxythiamine (OXY) was found to inhibit proliferation of tumor cells [34–36], the cytotoxicity assay for different doses of OXY in cultured human fibroblasts was performed. Cell viability was measured by the method of Carmichael et al. [26] using tetrazolium salt. The viability of OXY-treated fibroblasts is presented in Fig. 1a. OXY at studied concentrations did not influence the viability of the cells at 24 and 48 h incubation.

Collagen biosynthesis and prolidase activity were measured in confluent human dermal fibroblasts that have been treated with 30, 100, 300, and 1,000 µM of OXY. As can be seen in Fig. 1b, 24 and 48 h incubation of confluent fibroblasts in the medium containing 10 % of FBS and different concentrations of OXY contributed to increase in collagen biosynthesis in a dose-dependent manner. 1,000 µM OXY-induced increase in collagen biosynthesis by about 36 % of control, after 24 h incubation and by about 14 % of control after 48 h (Fig. 1b).

The mechanism of increase in collagen biosynthesis in OXY-treated cells may be due to the accumulation of pyruvate in the cell [2, 3]. This led us to investigate the influence of pyruvate on this process. Unexpectedly, we found that an addition of different concentrations of pyruvate (1, 3, 10, 20 mM) contributed to reduce in collagen biosynthesis. At concentration of 20 mM, pyruvate-induced decrease in collagen biosynthesis to about 50 % of control, after 24 h incubation (Fig. 1c).

**Fig. 2:**
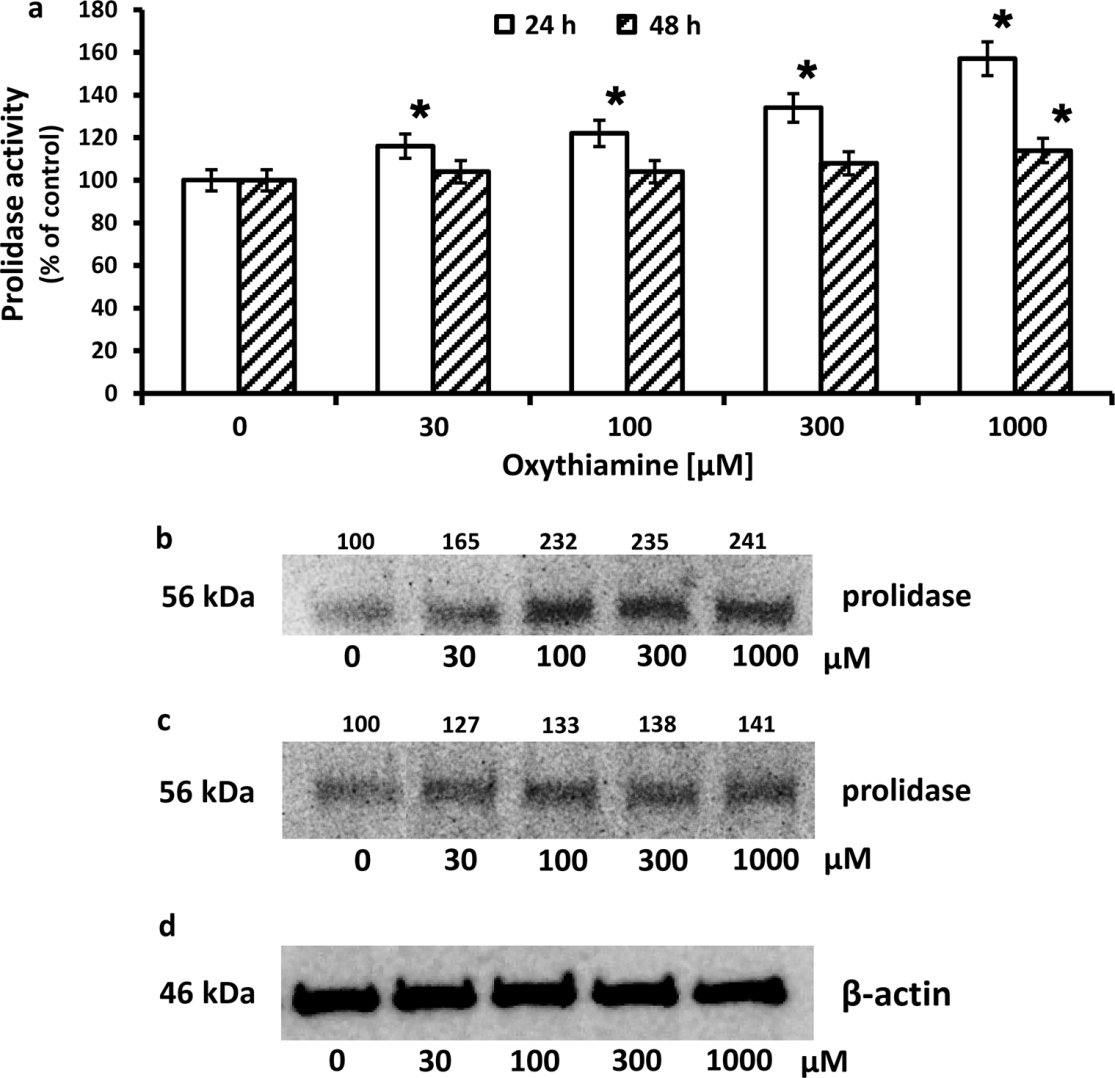
Prolidase activity **a** in confluent human skin fibroblasts incubated in the medium containing 10 % FBS and different concentrations of OXY. The results present the mean values from 6 assays ± SD *P ≤ 0.01. Western blot analysis for prolidase in fibroblasts incubated 24 h (**b**) and 48 h (**c**) with different concentrations of OXY. The mean values of six pooled cell homogenate extracts from six separate experiments are presented. The intensity of the bands was quantified by densitometric analysis. Densitometry was done with BioSpectrum Imaging System and presented as arbitrary units above the bands. 20 μg of supernatant protein was run in each lane for prolidase and β-actin. The expression of β-actin served as a control for protein loading (**d**)

To explain the mechanism of OXY-dependent increase in collagen biosynthesis, we considered prolidase as a target enzyme. Increase in collagen biosynthesis in OXY-treated cells was accompanied by increase in prolidase activity by about 57 and 14 % after 24 h and 48 h incubation, respectively, compared to control (Fig. 2a). An increase in prolidase activity was accompanied by increase in the level of the enzyme protein, by about 141 and 41 % after 24 and 48 h incubation, respectively, compared to control, as shown by Western immunoblot analysis (Fig. 2b, c).

The data show that OXY increases collagen biosynthesis in skin fibroblasts and suggest that an increase may result from stimulation of prolidase activity and expression.

We considered whether pyruvate or PEP is involved in OXY-dependent regulation of collagen biosynthesis. 3-MPA is an inhibitor of phosphoenolpyruvate carboxykinase (PEPCK). The enzyme is responsible for the conversion of oxaloacetate to PEP. We decided to check if inhibition of PEP formation can cause up-regulation of collagen biosynthesis and prolidase activity. In previous studies, we found that PEP down-regulate β_1_ integrin and IGF-I receptors signaling providing mechanism for inhibition of collagen biosynthesis in fibroblast. Moreover, we found that there is no direct correlation between collagen biosynthesis and prolidase activity and the enzyme level [19].

Therefore, the question was addressed whether an inhibitor of PEPCK, 3-mercaptopicolinate (3-MPA) affects oxythiamine-induced prolidase activity. As described previously, prolidase activity is stimulated by serum-derived growth factors [37]. Therefore, activity of this enzyme was measured in confluent fibroblasts, cultured for 1, 6, and 24 h in the medium containing 0.1 % of fetal bovine serum (FBS), 1 mM of 3-MPA, and different concentrations of OXY. The results were compared to the cells cultured for 1 h, 6 h, and 24 h in the medium containing 10 % FBS, 1 mM 3-MPA, and OXY.

**Fig. 3:**
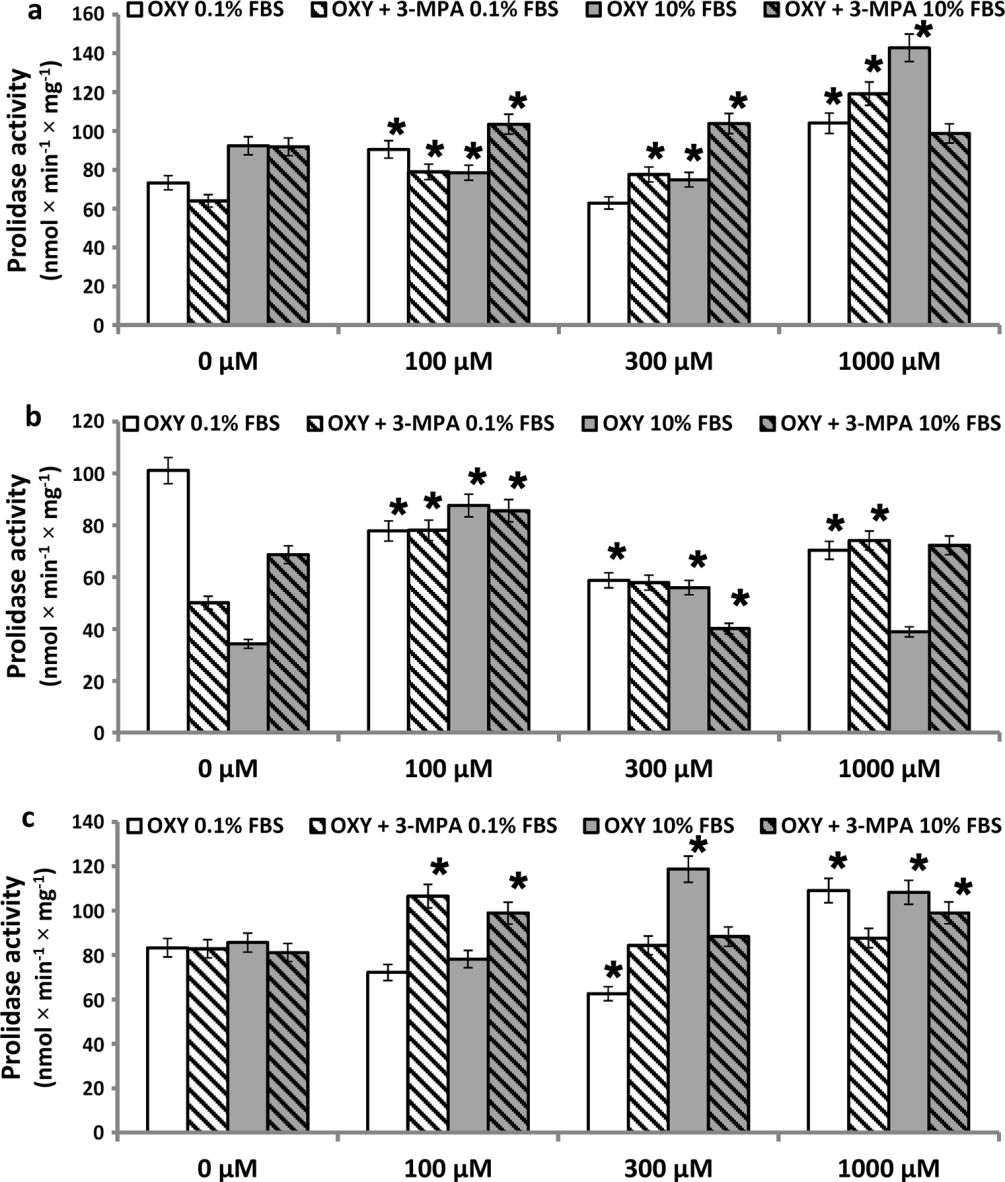
Prolidase activity in confluent human skin fibroblasts incubated for 1 h (**a**), 6 h (**b**) and 24 h (**c**) in the medium containing 0.1 % FBS or 10 % FBS and 1 mM of 3-mercaptopicolinate (3-MPA), and different concentrations of OXY. The results present the mean values from 6 assays ± SD *P ≤ 0.01

As can be seen in Fig. 3a and c, 1 and 24 h incubation of fibroblasts in the medium containing 0.1 % FBS and 1 mM OXY-induced prolidase activity, by about 40 and 30 %, respectively. However, 6 h incubation under the same conditions contributed to decrease in prolidase activity to 70 % of control (Fig. 3b). The mechanism of this phenomenon may involve PEP accumulation that inhibits prolidase activity and collagen biosynthesis.

An addition of 1 mM of 3-MPA under the same conditions contributed to increase in prolidase activity by about 86 and 48 % at 1 and 6 h incubation, and had no effect on prolidase activity after 24 h incubation. It may suggest that decreased activity of PEPCK contributes to suppression of PEP production (competitive inhibitor of prolidase activity) [20] and may be responsible for increase in the enzyme activity in first 6 h of incubation (Fig. 5, graphical abstract).

As can be seen in Fig. 3a and c, 1 and 24 h incubation of fibroblasts in the medium containing 10 % FBS and 1 mM OXY-induced prolidase activity, by about 55 and 27 % of control, respectively. After 6 h incubation, 100, 300, and 1,000 µM OXY increased prolidase activity by about 155, 63, and 13 % of control, respectively (Fig. 3b).

An addition of 1 mM of 3-MPA under the same conditions had small effect on prolidase activity after 1 and 6 h of incubation (increase by 8 and 5 % of control values, Fig. 3a, b) and contributed to increase in enzyme activity by about 22 % after 24 h incubation. In such conditions, we observed that PEPCK decreased prolidase activity (increased by OXY) after 1, 6, and 24 h incubation. It suggests that decreased activity of PEPCK contributes to suppression of PEP production (competitive inhibitor of prolidase activity) [20] and is responsible for slight increase in the enzyme activity (Fig. 5, graphical abstract).

**Fig. 4:**
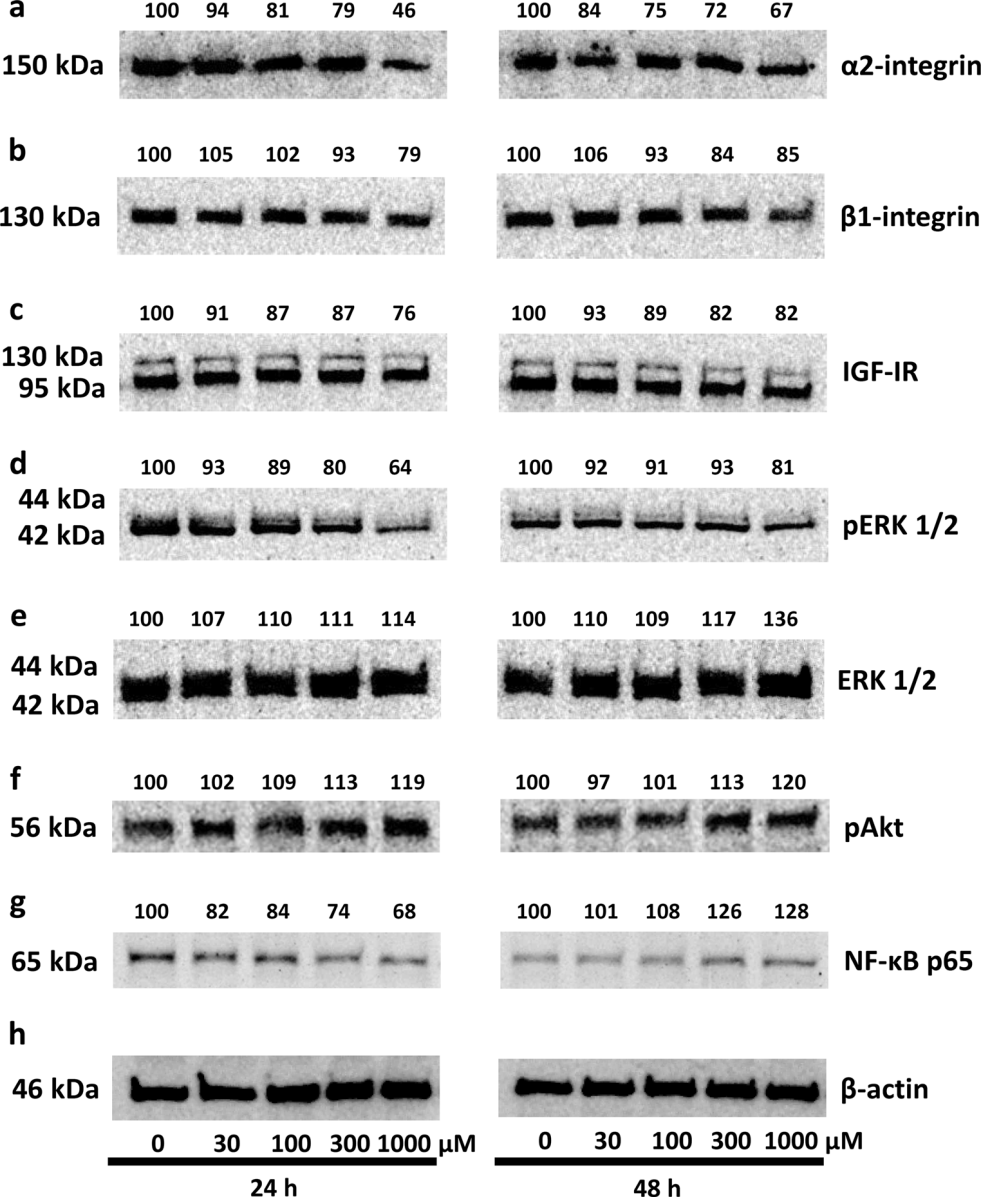
Western blot analysis for α_2_-integrin (**a**), β_1_-integrin (**b**) receptor subunits, IGF-I receptor (**c**), pERK1/2 (**d**), ERK1/2 (**e**), pAkt (**f**), and NF-*κ*B p 65 (**g**) in control human skin fibroblasts and cultured for 24 and 48 h in the medium containing different concentrations of OXY. The mean values of six pooled cell homogenate extracts from six separate experiments are presented. The intensity of the bands was quantified by densitometric analysis. Densitometry was done with BioSpectrum Imaging System and presented as arbitrary units b. The same amount of supernatant protein (20 μg) was run in each lane. The expression of β-actin served as a control for protein loading (**h**)

Collagen biosynthesis and prolidase activity were previously shown to be regulated due to the signal induced by activated α_2_β_1_ integrin receptor [17] as well as IGF-IR [12, 24]. Therefore, the expression of α_2_β_1_ integrin receptor (receptor for type I collagen) and IGF-IR was measured by Western immunoblot analysis. As can be seen in Fig. 4a, 24 and 48 h treatment of fibroblasts with 30, 100, 300 and 1,000 µM of OXY contributed to a distinct decrease in the expression of α_2_ integrin subunit to about 94, 81, 79, and 46 % respectively, at 24 h, and to about 84, 75, 72, and 67 %, respectively, at 48 h, compared to the control cells. 24 and 48 h treatment of fibroblasts with 1,000 µM of OXY reduced expression of β_1_ integrin subunit to about 79 and 85 %, respectively, compared to the control (Fig. 4b). As shown in Fig. 4c, IGF-I receptor expression was decreased for 1,000 µM OXY-treated cells to about 76 and 82 % at 24 and 48 h incubation, respectively, compared to the control cells. We found that the highest concentration of OXY decreases expression of phosphorylated ERK1/2 to about 64 and 81 % at 24 and 48 h incubation, respectively (Fig. 4d) and stimulates expression of total ERK1/2 by about 14 % at 24 h and by about 36 % at 48 h, compared with control (Fig. 4e). Simultaneously, 1,000 µM of OXY-dependent stimulation of total ERK1/2 expressions (Fig. 4e) was accompanied by similar induction of phosphorylation of Akt by about 19 and 20 % at 24 and 48 h (Fig. 4f), suggesting that in the experimental conditions, ERK1/2 and Akt proteins represent signaling molecules that respond to OXY action.

On the other hand, we have found that in 24 h OXY-treated cells there is decrease in the expression of NF-*κ*B p65, the known inhibitor of collagen gene expression [25], compared to control cells (Fig. 4g). Opposite phenomenon was observed after 48 h incubation with OXY. An increase in the expression of NF-*κ*B p65 can explain only slight increase in collagen biosynthesis due to OXY action at this time (Fig. 1b).

In view of this data, it seems that OXY-dependent increase in collagen biosynthesis may be a consequence of increase in prolidase activity and the enzyme protein level, increase in pAkt level, and inhibition of pERK1/2 and NF- κB p65.

## Discussion

Skin fibroblasts are connective tissue cells specialized in collagen biosynthesis. For this reason, they are a good model for studies of the effects of regulatory factors on collagen metabolism. Furthermore, fibroblasts would undergo aerobic glycolysis providing increased pyruvate/lactate that could then be used for the mitochondrial TCA cycle, oxidative phosphorylation, and ATP production for anabolic stimulation.

The data presented here show that OXY induces an increase in collagen biosynthesis in human skin fibroblasts. The increase in collagen biosynthesis was correlated with an increase in prolidase activity and the enzyme protein level. Our studies support the relationship between collagen synthesis and prolidase activity. A similar relationship was observed during the fibrotic process, where an increase in prolidase activity was accompanied by an increase in tissue collagen deposition [12]. The link between collagen production and prolidase activity was also found in cultured human skin fibroblasts treated with anti-inflammatory drugs [13], during experimental aging of these cells [14], fibroblasts chemotaxis [37] and cell surface integrin receptor ligation [17]. However, the link between OXY-dependent accumulation of pyruvate and up-regulation of collagen biosynthesis is not fully understood. Previously, we proposed that the mechanism may originate at the level of interconversion of pyruvate/PEP.

Oxythiamine (OXY) affects pentose phosphate pathways [35] and by inhibition of pyruvate decarboxylase increases the amount of pyruvate [2]. Pyruvate is converted into PEP by PEPCK catalyzing phosphorylation and concomitant decarboxylation of oxaloacetate. This reaction is the first step of the gluconeogenic pathway, which is subject to regulation. PEP is known as a strong, competitive inhibitor of prolidase activity “in vitro” [20]. In our previous study, we found that PEP contributed to decrease in collagen biosynthesis in cultured human skin fibroblasts through depression of α_2_β_1_ integrin and IGF-IR signaling. However, we suggest that there is no direct correlation between collagen biosynthesis and prolidase activity and level [19].

**Fig. 5:**
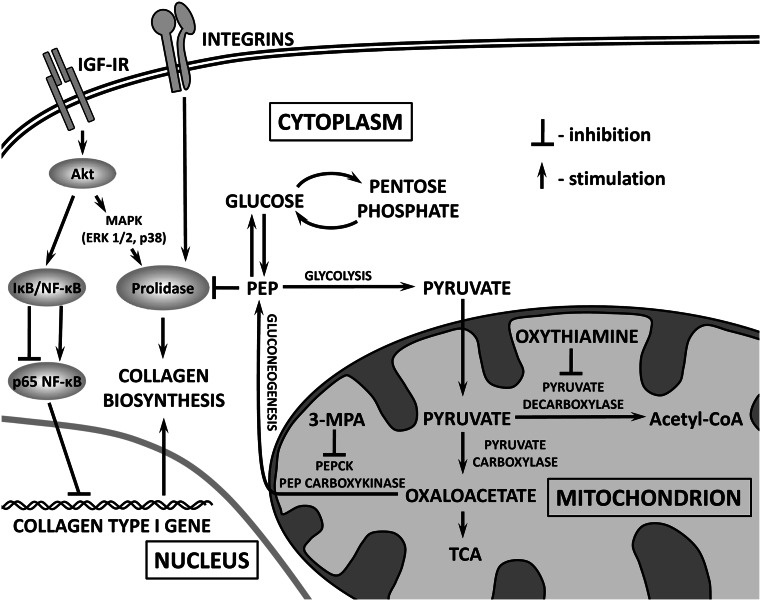
Graphical abstract. In the experimental conditions (OXY-treated cells for 24 h), there is up-regulation of AKT that contributes to stimulation of collagen biosynthesis through activation of prolidase—an enzyme supporting proline for collagen biosynthesis and down-regulation of NF-kB—transcription factor that inhibits collagen type I gene expression. During that time, OXY directs pyruvate for TCA cycle that eliminate gluconeogenesis and production of PEP—inhibitor of prolidase activity. The net result is up-regulation of collagen biosynthesis. However, 48 h incubation of the cells with OXY may contribute to accumulation of oxaloacetate in mitochondria and stimulation of gluconeogenesis that increases cytoplasmic concentration of PEP—inhibitor of prolidase and consequently collagen biosynthesis. This process may be augmented by inhibitory effect of OXY on pentose cycle

We investigated whether pyruvate or PEP is involved in OXY-dependent regulation of collagen biosynthesis. It is apparent from our study that inhibitor of PEPCK up-regulated prolidase activity in different experimental conditions. It suggests that decreased activity of PEPCK contributes to suppression of PEP production (competitive inhibitor of prolidase activity) [20] and is responsible for slight increase in the enzyme activity. In certain experimental conditions (6 h incubation), we found OXY-dependent inhibition of prolidase activity resulted probably from PEP accumulation. The potential mechanism of OXY-dependent regulation of prolidase activity and collagen biosynthesis is outlined in Fig. 5.

Collagen is known as a ligand for α_2_β_1_ integrin. Previously, it has been shown that β_1_-integrin receptor is involved in signaling, which regulates collagen biosynthesis [38] and prolidase activity [17, 39]. Another important point of collagen biosynthesis regulation is at the level of insulin-like growth factor-I receptor (IGF-IR). IGF-I is one of the most potent collagen-stimulating factors in collagen synthesizing cells [22]. Therefore, we considered β_1_ integrin and IGF-IR as a potential target in OXY-induced increase of the above processes.

Our observations suggest that in fibroblasts OXY induces collagen biosynthesis independently of α_2_β_1_ integrin receptor and IGF-IR expressions. Presumably, increase in prolidase activity and level in fibroblasts due to OXY action is a result of increase in ERK1/2 signaling and stimulation of Akt, suggesting that in experimental conditions ERK1/2 and Akt proteins represent signaling molecules that respond to OXY action.

Several other studies suggest that prolidase-dependent regulation of collagen biosynthesis may take place at the transcriptional level. The transfection of colorectal cancer cells with prolidase vector inhibited NF-κB p65 expression [40], well-recognized inhibitor of expression of α1 and α2 subunits of type I collagen [25, 41, 42]. Another evidence for the role of prolidase in regulation of NF-κB expression provides experiment showing that inhibition of prolidase activity by Cbz-Pro contributed to up-regulation of NF-κB p65 expression in fibroblasts [40]. In fact, our data showed that OXY-dependent increase in prolidase activity (by about 57 % of control) and collagen biosynthesis (by about 36 % of control) is accompanied by decrease in the expression of NF-κB p65, after 24 h incubation. During 48 h incubation of fibroblasts in medium, supplemented with OXY prolidase activity and collagen biosynthesis was not significantly increased (both by about 14 % of control) and down-regulation of NF-*κ*B p65 was not observed.

Although the link between up-regulation of AKT and down-regulation of ERK1/2 and NF-κB in OXY-treated cells is not known, it cannot be excluded that underlying mechanism may also involve pentose cycle. OXY is known as a transketolase inhibitor that may cause a severe deficiency in high-energy phosphate bonds, contributing to decrease in protein phosphorylation and cell cycle arrest [36].

It has been demonstrated in many in vitro studies that OXY inhibits cancer cell growth through suppression of the cell cycle. However, the molecular mechanism of the action is poorly understood. Some data suggested that in pancreatic cancer cells OXY may specifically induce cell cycle arrest through deactivation of the MAPK pathways, without effects on NF-κB [43]. Whether such a mechanism takes place in normal cells requires to be explored.

The data suggest that OXY may exert its effect on collagen biosynthesis through stimulation of prolidase activity and the enzyme protein level, pAkt level, and inhibition in pERK1/2 and NF- κB p65 transcriptional activity.
